# Toward a molecular mechanism for the interaction of ATP with alpha-synuclein†

**DOI:** 10.1039/d3sc03612j

**Published:** 2023-08-26

**Authors:** Evelyn Rose Kamski-Hennekam, Jinfeng Huang, Rashik Ahmed, Giuseppe Melacini

**Affiliations:** a Department of Chemistry and Chemical Biology, McMaster University Hamilton ON L8S 4M1 Canada melacin@mcmaster.ca; b Department of Biochemistry and Biomedical Sciences, McMaster University Hamilton ON L8S 4M1 Canada

## Abstract

The ability of Adenosine Triphosphate (ATP) to modulate protein solubility establishes a critical link between ATP homeostasis and proteinopathies, such as Parkinson's (PD). The most significant risk factor for PD is aging, and ATP levels decline dramatically with age. However, the mechanism by which ATP interacts with alpha-synuclein (αS), whose aggregation is characteristic of PD, is currently not fully understood, as is ATP's effect on αS aggregation. Here, we use nuclear magnetic resonance spectroscopy as well as fluorescence, dynamic light scattering and microscopy to show that ATP affects multiple species in the αS self-association cascade. The triphosphate moiety of ATP disrupts long-range electrostatic intramolecular contacts in αS monomers to enhance initial aggregation, while also inhibiting the formation of late-stage β-sheet fibrils by disrupting monomer–fibril interactions. These effects are modulated by magnesium ions and early onset PD-related αS mutations, suggesting that loss of the ATP hydrotropic function on αS fibrillization may play a role in PD etiology.

## Abstract 2

We propose a mechanism for the hydrotrope-like function of ATP on alpha-synuclein whereby ATP enhances initial αS aggregation and inhibits fibril formation in a phosphate-dependent manner that is modulated by PD-related αS mutations E46K and A53T.

## Graphical abstract

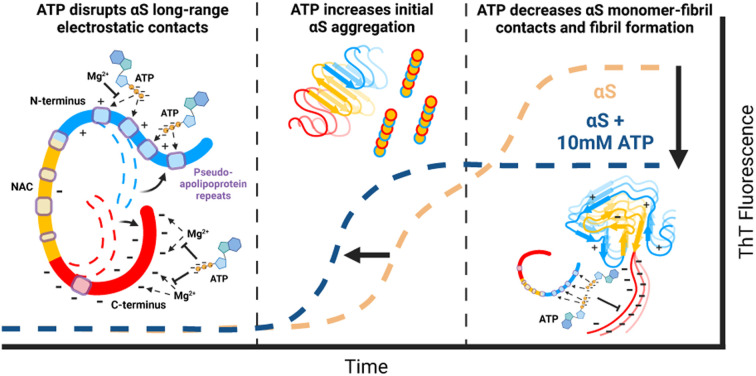

## Introduction

All cells maintain physiological concentrations of Adenosine Triphosphate (ATP) in the 1–12 mM range, significantly higher than what is required for ATP to provide energy or serve as a phosphate source in phosphorylation processes.^1–5^ Instead, the mM levels of ATP critically allow ATP to bind aggregation-prone proteins and modulate their solubility.^3–8^ One protein for which this modulatory effect of ATP is particularly relevant – yet currently unclear – is alpha-synuclein (αS), whose aggregation is closely linked to the pathology of Parkinson's Disease (PD).^9^

Age is the primary risk factor for idiopathic PD, with incidence of PD increasing from ∼1% for individuals over 60 to 5% for those over 85.^10^ Interestingly, aging is also accompanied by a dramatic decline of ATP levels in multiple model organisms.^11^ Older *Caenorhabditis elegans* and *Drosophila* exhibit ATP levels approximately 80% and 50% lower than their younger counterparts, respectively.^11^ In agreement with these observations, ATP levels decrease with age in the mouse brain, as well as in the cardiac muscles of both mice and humans.^11^ Given the age-related parallels between increasing PD prevalence and decreasing ATP levels, it is possible that a modulating effect of ATP on αS aggregation could be a factor in PD onset.^12^

Indeed, *in vivo* studies show that adenine supplementation to increase ATP levels from 1 to 4 mM significantly reduces the number of αS-GFP-positive foci/aggregates in yeast.^13^ Additionally, yeast strains with significantly reduced ATP levels show increased sensitivity to αS-GFP, as measured by reduced growth, suggesting that higher ATP levels likely reduce αS cytotoxicity.^13^ Therefore, the available data collectively suggest that ATP likely elicits a physiologically- and pathologically-relevant effect on αS aggregation.^14,15^ However, the mechanism by which ATP exerts this effect is currently unclear.

**Fig. 1:**
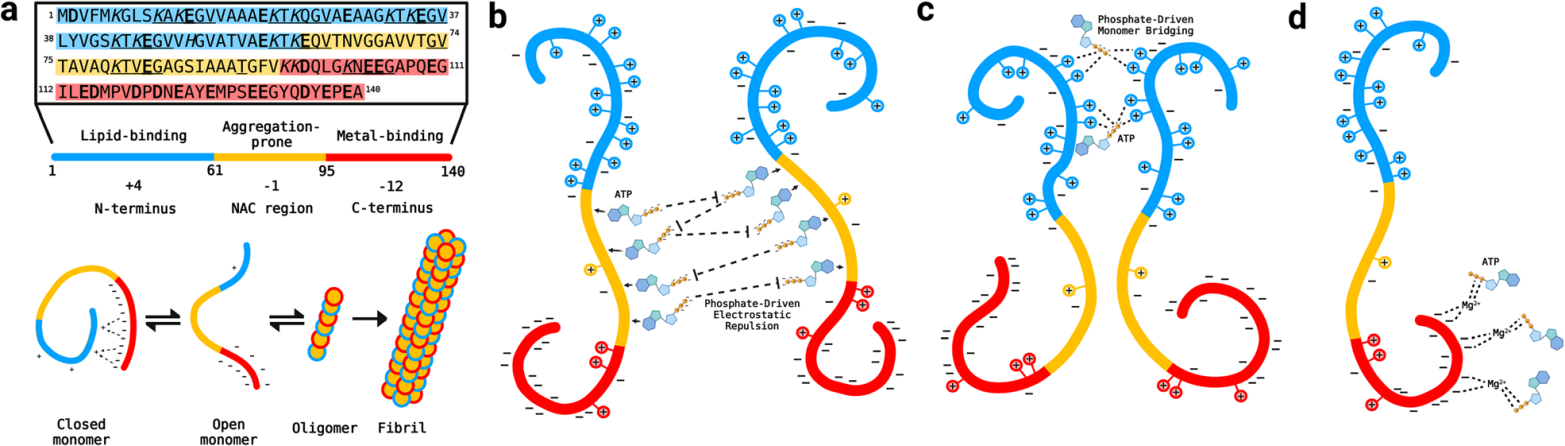
Hypothetical models of αS–ATP interactions. (a) Upper panel: WT αS amino acid sequence, containing nine pseudo-apolipoprotein-like repeats (underlined), acidic (bolded) and basic (italicized) residues.^17^ The N-terminus, NAC region and C-terminus are colored in blue, yellow and red, respectively.^16^ Lower panel: general αS aggregation mechanism involving disruption of the long-range N- to C-terminal monomer contacts, leading to opening and subsequent aggregation of αS into oligomers and fibrils.^21–24^ (b) One hypothetical model of the ATP–αS interaction based upon the interaction of ATP with proteins in the crystalline lens, whereby the adenine group of ATP clusters over protein hydrophobic patches and the triphosphate electrostatically repels other bound monomers to inhibit aggregation.^25^ (c) Another viable model for ATP–αS interactions, suggesting that ATP could bridge αS monomers and enhance aggregation *via* the phosphate-mediated targeting of lysine residues (designated by “+” symbols) in the αS N-terminus, as is the case for tau protein.^6^ (d) Model for Mg^2+^-mediated ATP–αS interaction proposed by Nishizawa *et al.*, whereby Mg^2+^ ions interact with the αS C-terminus and “bridge” indirect, non-specific interactions between the protein and ATP.^12^

Alpha-synuclein is a 140-amino-acid protein divided into three regions with distinct net charges (Fig. 1a): a positively-charged N-terminus (residues 1–60) rich in lysine residues, a predominantly hydrophobic, non-amyloid-β component or NAC region (residues 61–95) that drives aggregation and an acidic C-terminus (residues 96–140) that binds a variety of metal ions.^16,17^ Throughout the αS sequence the consensus motif “KTKEGV” is repeated nine times either completely or partially (Fig. 1a) and these pseudo-apolipoprotein-like repeats confer lipid-binding properties to αS.^17,18^ However, under pathological conditions αS monomers form insoluble, β-sheet-rich amyloid fibrils as well as neurotoxic oligomeric intermediates.^18^

Many factors influence αS aggregation, including αS point mutations such as E46K and A53T, which are associated with familial PD and cause disease onset decades earlier than under idiopathic conditions.^19^ In addition, tetra-polyphosphates interact electrostatically with the “KTK” segments of the N-terminal “KTKEGV” repeats of αS monomers to enhance charge-driven aggregation by disrupting long-range electrostatic contacts between the N- and C-termini of αS monomers.^20^ These contacts shield the aggregation-prone NAC region of αS monomers and thereby inhibit aggregation (Fig. 1a; closed monomer).^21^ Consequently, perturbation of these contacts by charged species, including heparin, spermine and Na^+^, leads to exposure of the NAC region, allowing it to establish intermolecular interactions that drive pathological oligomer and fibril formation (Fig. 1a).^21–24^

It is currently unclear to what extent the mechanisms of polyphosphate-αS interactions can be transferred to ATP–αS complexes. A model proposed to explain the hydrotrope-like effect of ATP on aggregation-prone proteins suggests that the aromatic purine ring of ATP clusters over protein hydrophobic patches while the triphosphate chain interacts with bulk water to prevent the formation of aggregates by repelling other such bound monomers (Fig. 1b).^25^ However, ATP can also elicit a pro-aggregation effect on positively-charged amyloidogenic proteins, *e.g.*, tau and human muscle acylphosphatase, by electrostatically binding to lysine residues of multiple monomers to enhance nucleating dimer formation (Fig. 1c).^6,7^ Similar interactions have been reported for the complexes formed by ATP and proteins undergoing phase separation. Phase separation of Fused-In-Sarcoma (FUS) protein is modulated by the adenine ring of ATP forming π–π interactions with aromatic side chains, while the triphosphate interacts electrostatically with arginine and lysine residues.^8^ The ATP : protein ratio is also critical for determining the effect of ATP, as ratios less than 100 : 1 promote phase separation of TDP-43 and FUS proteins *via* bivalent binding, while ratios above 100 : 1 elicit the opposite effect.^5,8^ Finally, Nishizawa *et al.* proposed that ATP interacts indirectly with αS *via* weak, non-specific interactions that are driven by Mg^2+^ serving as a bridge between ATP and the αS C-terminus (Fig. 1d).^12^

While these studies provide important clues on αS–ATP complexes, several questions remain open on how ATP interacts with αS and how these interactions are modulated by pathologically-relevant factors, such as PD-related αS mutations. The effect of ATP on αS monomer conformations and on pathologically-relevant αS aggregation is also still unexplored. Addressing these gaps is critical to understanding the physiological and pathological roles of ATP.

Here, we probe how ATP interacts with multiple αS species and how these interactions modulate αS intramolecular and monomer–fibril contacts and aggregation. By complementing multiple Nuclear Magnetic Resonance (NMR) experiments with Thioflavin T (ThT) fluorescence, dynamic light scattering (DLS) and microscopy, we explore the effect of ATP on both early- and late-stage αS aggregation. We show that ATP causes an enhancement of early αS aggregation by disrupting long-range electrostatic contacts in αS monomers and thereby shortening the lag time for β-sheet fibril formation. Strikingly, elevated ATP levels also significantly inhibit late-stage αS aggregation as well as N-terminally-driven αS monomer–fibril contacts that are critical for templated fibril elongation and pathologically-relevant secondary nucleation.^9,26^ We also show that the triphosphate moiety of ATP drives its primarily electrostatic interaction with the lysine- and threonine-dense “KTKEGV” N-terminal repeats in αS monomers.^17,18^ These ATP–αS interactions are modulated by Mg^2+^, which sequesters ATP from αS as ATP-Mg and *vice versa*, as well as by αS mutations that perturb the lysine- and threonine-distributions (E46K and A53T). Our data reveal that the E46K and A53T αS mutations dramatically alter the effect of ATP on αS aggregation, causing a significant increase in the population of soluble aggregates of intermediate-size. Overall, these results provide a mechanism to begin understanding the role of ATP in αS-related PD-pathophysiology.

## Results and discussion

### ATP elicits a concentration-dependent effect on αS aggregation that is phosphate-dependent and is preserved in the presence of Mg^2+^

**Fig. 2:**
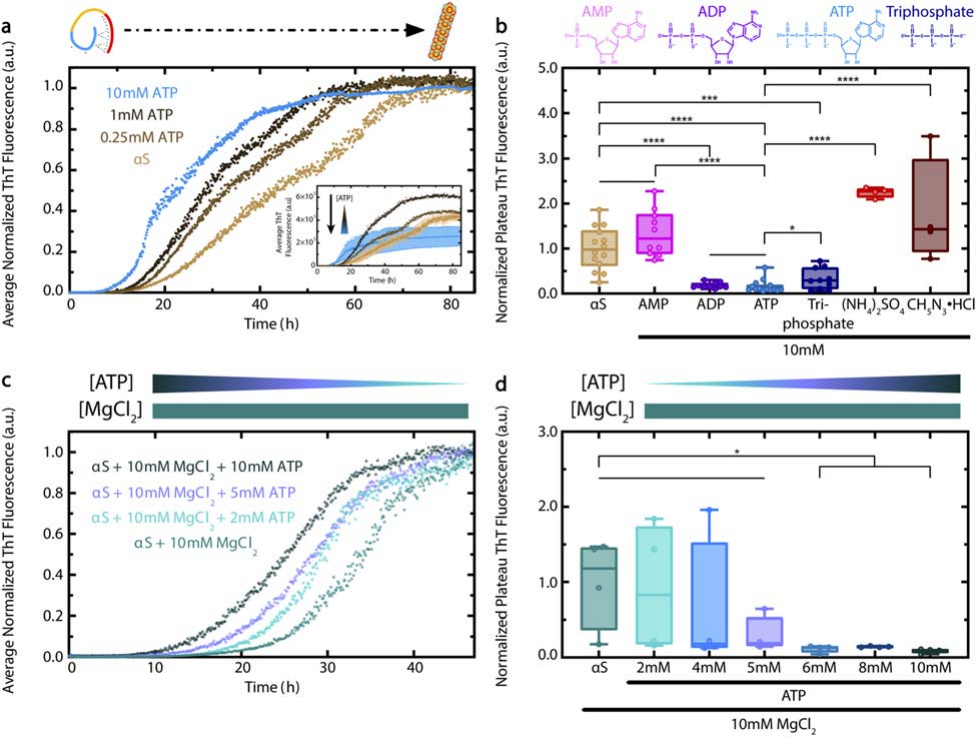
ATP elicits a concentration-dependent effect on αS aggregation that is phosphate-driven and is preserved in the presence of magnesium. (a) ThT fluorescence of fresh 300 μM WT αS in ThT buffer (20 mM K_2_HPO_4_, 5 mM KH_2_PO_4_, 100 mM KCl, 200 μM EDTA, 0.05% NaN_3_), incubated for 85 h in a 37 °C plate reader. Plotted are average ThT measurements across four wells per condition, with measurements taken every six minutes with 30 s orbital shaking prior to each read and normalized to the final measurement for the αS sample. Inset shows non-normalized data, with standard deviations for the αS and αS + 10 mM ATP conditions shown. Error bars for ATP < 10 mM are not shown to avoid overcrowding, but are comparable to 10 mM ATP. (b) ThT fluorescence of fresh 300 μM WT αS in ThT buffer, incubated for 72 h in a 37 °C shaker at 150 rpm then in a plate reader for 20 additional hours at plateau. Plotted are well-specific average ThT measurements from multiple independent experiments, taken at plateau every five min with 30 s orbital shaking prior to each read and normalized to the average measurement of all αS samples. Ligand concentrations are all 10 mM. Chemical structures of AMP, ADP, ATP and triphosphate at pH 7.4 are shown above the panel. (c) ThT fluorescence of fresh 440 μM WT αS in ThT buffer, incubated for 47 h in a 37 °C plate reader. Plotted are average ThT measurements across four wells per condition, with measurements taken every six minutes with 30 s orbital shaking prior to each read and normalized to the final measurement of the αS + 10 mM MgCl_2_ sample. (d) Well-specific average ThT fluorescence measurements of panel (c) samples at plateau (40–47 h), normalized to the average measurement of all αS + 10 mM MgCl_2_ samples. Straight lines between samples in panels (b) and (d) represent no significant difference. Sample comparisons in panels (b) and (d) represent significance levels: * = *p* < 0.05, *** = *p* < 0.001 and **** = *p* < 0.0001.

To determine the effect of ATP on αS aggregation, we measured ThT fluorescence of Wild-Type (WT) αS incubated with increasing concentrations of ATP.^9,16–18^ We found that ATP causes a concentration-dependent shortening of the lag time for αS aggregation (Fig. 2a). This effect is already evident at ATP concentrations as low as 0.25 mM, and is particularly significant at 10 mM. Interestingly, the aggregation-accelerating effect of ATP on αS is similar yet distinct from the effect of polyphosphates, as ATP causes a significant shortening of the αS lag time at much lower concentrations than do tri-polyphosphates.^20^ In addition, 10 mM ATP causes a statistically-significant reduction of αS ThT fluorescence at plateau that is different from the enhancements caused by 0.25 and 1 mM ATP (Fig. 2a inset). The decreased plateau ThT fluorescence observed in the presence of 10 mM ATP reflects a loss in the amount of late-stage αS aggregates/fibrils, as confirmed by Transmission Electron Microscopy (TEM) (ESI Fig. S1†). Furthermore, comparative DLS analyses show that 10 mM ATP also reduces the formation of soluble, intermediate-size αS aggregates at plateau (ESI Fig. S2†). The ability of 10 mM ATP to both enhance early αS aggregation by shortening the lag time and concurrently inhibit αS ThT fluorescence at plateau reveals a unique bi-phasic effect of ATP on protein aggregation. Overall, our ATP- and time-dependent ThT data provide an initial explanation for how ATP influences αS aggregation.

Since the formation of mature αS fibrils is a hallmark of PD pathology, we next sought to determine the driving force for the ATP-dependent modulation of αS ThT fluorescence at plateau.^9^ To this end, we compared the effect of 10 mM ATP on αS to that of ATP analogs which differ from ATP in the number of phosphate groups, *i.e.*, Adenosine Monophosphate (AMP) and Adenosine Diphosphate (ADP). Interestingly, ADP – but not AMP – is able to significantly reduce αS ThT fluorescence at plateau to a similar extent as ATP, suggesting that ATP's effect on αS at plateau requires two or more phosphate groups (Fig. 2b). Additionally, the triphosphate-mediated reduction of αS ThT fluorescence at plateau is statistically different from that of ATP, suggesting that the observed effect of ATP on αS aggregation is not completely recapitulated by another small molecule with three negative phosphate groups (Fig. 2b). In addition, the effects of ammonium sulfate and guanidine hydrochloride, which were selected as they are at opposite ends of the Hofmeister series, are significantly different than that of ATP (Fig. 2b). From this we conclude that the effect of ATP on pathologically-relevant late-stage αS fibril formation is not likely a general salting effect. Given the importance of the triphosphate moiety, the effect of ATP is instead likely to specifically require nucleotides and not inorganic salts (Fig. 2b).^9^ In this respect, the interaction between ATP and αS shares some common features with that between ATP and lysozyme, which was recently modelled by Ou *et al.* using MD simulations.^27^ Ou *et al.* found that although the phosphate groups of ATP tend to form salt bridges primarily with positively-charged lysozyme residues, the adenine and ribose moieties of bound ATP molecules also form hydrogen bonds with the protein.^27^

We next explored whether ATP is still able to shorten the lag time for αS aggregation and reduce its plateau ThT fluorescence in the presence of equimolar Mg^2+^, since Mg^2+^ complexation is required for ATP's biological function as an energy source.^28,29^ In addition, Mg^2+^ levels decline with age and are often greatly reduced in PD patients.^7,30^ We observed that ATP can still cause a concentration-dependent decrease in the αS lag time, even in the presence of high Mg^2+^ (Fig. 2c). ATP concentrations above 5 mM also cause a significant reduction in αS ThT fluorescence at plateau (Fig. 2d). Thus, the bi-phasic effect of ATP on αS aggregation persists in the presence of high Mg^2+^ concentrations, suggesting that Mg^2+^ is unable to silence the effect of ATP on αS aggregation.

As αS cytotoxicity correlates inversely with aggregate size, it is likely that the effects of ATP on αS aggregation in both the presence and absence of Mg^2+^, as well as the altered levels of ATP and Mg^2+^ with increasing age, play a pleiotropic role in PD pathology.^9,11,13,31^ To understand the mechanisms underlying the effect of ATP on αS aggregation, we turned to NMR to characterize the interactions between ATP and αS and to evaluate how these interactions are modulated by Mg^2+^.

### The triphosphate group of ATP drives electrostatic interactions with N-terminal lysine and threonine residues in αS monomers

**Fig. 3:**
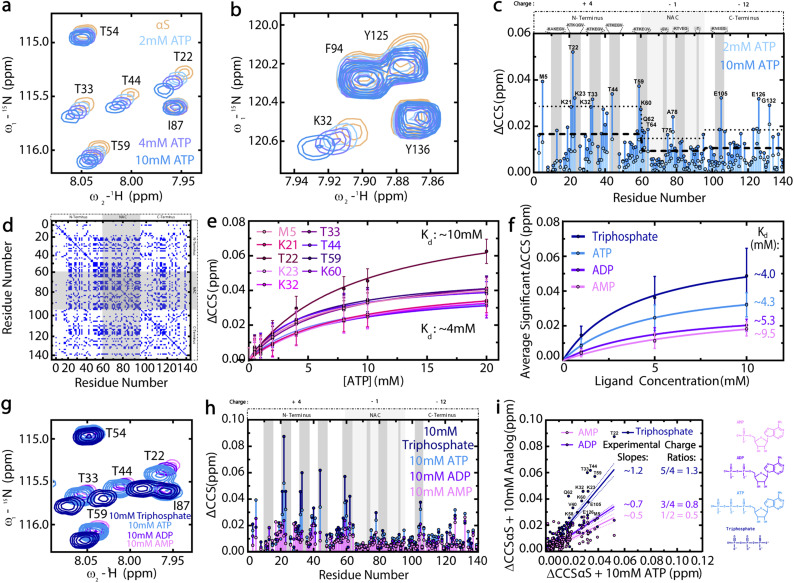
The triphosphate moiety of ATP drives electrostatic interactions between ATP and the N-terminal pseudo-apolipoprotein repeats of WT αS. (a and b) ^1^H–^15^N HSQC spectral regions of WT αS monomers with increasing ATP concentrations. (c) ATP-induced Compounded Chemical Shifts (ΔCCS) of WT αS monomers, with ΔCCS greater than the region-specific average shift (dashed line) plus one standard deviation (dotted line) labelled. (d) Cos *θ* cross-peaks > 0.97 for αS WT (“apo”) *versus* 10 mM ATP-bound αS WT (“holo”). (e) 0–20 mM ATP-induced ΔCCS of significantly-shifted N-terminal αS residues fitted to a one-site specific binding model.^38–40^ Upper and lower-limit model-calculated *K*_d_ values are shown. (f) Average significant αS ΔCCS induced by AMP, ADP, ATP or triphosphate, fitted to one-site specific binding models and with approximate fitted *K*_d_ values shown. Error bars represent the standard deviation of well-resolved peaks at each concentration. (g) HSQC spectral regions used to calculate panel (h) ΔCCS. (h) αS ΔCCS induced by 10 mM AMP, ADP, ATP or triphosphate. The HSQC spectrum of αS in the presence of 10 mM ATP and the resulting ATP-induced αS ΔCCS profile shown in (a) and (c) are reproduced in (g) and (h), respectively, for ease of comparison. αS net regional charges are shown above panels (c) and (h), with dark grey boxes representing the “KTKEGV” αS repeats.^17^ (i) Correlations between 10 mM ATP-induced αS ΔCCS and those induced by 10 mM AMP, ADP or triphosphate. Approximate slopes are shown with lines of best fit and errors. Structures of AMP, ADP, ATP and triphosphate at pH 7.4 are shown. ΔCCS were calculated as ΔCCS = (0.5*((δH_AMP, ADP, ATP or triphosphate_ − δH_αS_)^2^ + (0.15*(δN_AMP, ADP, ATP or triphosphate_ − δN_αS_)^2^)))^1/2^.^42^

We next characterized the driving mechanism for the ATP–αS interaction by measuring the ATP-induced ^1^H–^15^N HSQC-based chemical shifts of WT αS monomers. Fig. 3a–c show that ATP induces concentration-dependent shifts primarily in lysine and threonine residues within the N-terminal pseudo-apolipoprotein-like repeats of αS, leaving the NAC and C-terminal imperfect “KTKEGV” repeats largely unaffected and thereby suggesting a level of specificity for the ATP–αS interaction.^16,17^ Such specificity is notable as the binding of ATP to αS is weak with *K*_ds_ in the 4–10 mM range, as shown by the binding isotherms built using the most significant, N-terminal αS ATP-induced chemical shifts (Fig. 3e). Interestingly, these mM *K*_d_ values are similar to the reported range of cellular ATP concentrations, indicating that a significant proportion of cellular αS is likely bound to ATP.^4,5^

Another notable feature of the ATP-induced chemical shifts in Fig. 3a and b is their parallel pattern which is quantitatively confirmed by the cos *θ*_*ij*_ matrix in Fig. 3d and suggests that ATP approaches different αS residues in a consistent orientation.^32^ However, based on the chemical shift maps of Fig. 3a–c, it is clear that the high local concentrations of polar and positively-charged residues in the αS N-terminus is critical for the ATP–αS interaction and predominates over π–π stacking interactions since aromatic αS residues in the C-terminus and hydrophobic NAC region show less significant, concentration-dependent ATP-induced chemical shifts (Fig. 3b and c).^17^ These data rule out the hypothesis that the ATP–αS interactions are driven by the adenine ring of ATP clustering over protein hydrophobic patches (Fig. 1b), while ruling in other models in which ATP–αS binding is mediated primarily by the triphosphate (Fig. 1c).^6,7,25^

To confirm the hypothesis that the triphosphate moiety of ATP drives its electrostatic interaction with αS, we compared the αS chemical shifts induced by ATP to those induced by AMP, ADP and triphosphate (Fig. 3f–h). These ATP analogs cause phosphate-dependent shifts mainly in the same N-terminal threonine residues targeted by polyphosphates and result in higher average *K*_d_ values for ADP and AMP *vs.* ATP and triphosphate (Fig. 3f–h).^20^ In addition, linear correlations between the αS chemical shifts induced by ATP analogs *versus* ATP exhibit slopes comparable to the ratio of charges between the respective ATP analogs and ATP at physiological pH 7 (Fig. 3i), corroborating that charge is a primary driver of ATP binding to αS. Given this charge-dependence, we hypothesized that Mg^2+^ complexation with the phosphates of ATP and the corresponding partial charge neutralization of the triphosphate moiety would influence the ATP–αS interaction.^28^

### Magnesium modulates the interaction of ATP with αS monomers and *vice versa*

**Fig. 4:**
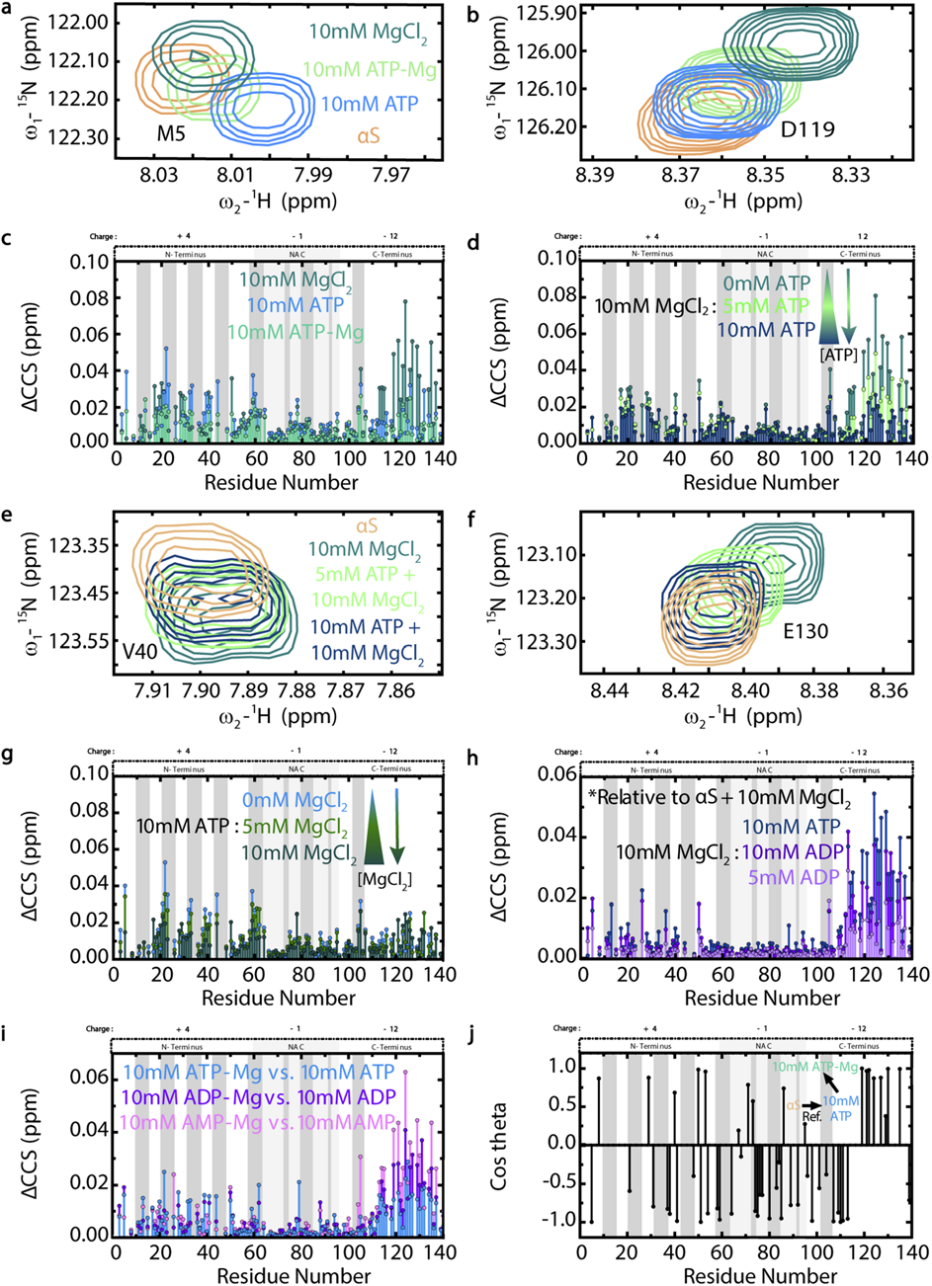
Magnesium modulates the ATP–αS monomer interaction and *vice versa*. (a and b) ^1^H–^15^N HSQC spectral regions of αS in the absence or presence of 10 mM ATP, ATP-Mg or MgCl_2_, colored as per legend in (a). (c) ΔCCS from panels (a) and (b) spectra. (d) αS ΔCCS calculated from panels (e) and (f) spectra, showing αS pre-incubated overnight with 10 mM MgCl_2_ or with the subsequent addition of 5 or 10 mM ATP. Panels (e) and (f) are colored as per legend in (e) and include the αS alone spectrum for reference. (g) 10 mM ATP-induced αS ΔCCS from pre-incubation overnight or with subsequent addition of 5 or 10 mM MgCl_2_. (h) 10 mM MgCl_2_-induced αS ΔCCS from pre-incubation overnight or with subsequent addition of 5 or 10 mM ADP or 10 mM ATP. ΔCCS for this panel calculated as: ΔCCS = (0.5*((δH_(ADP or ATP)+10 mM MgCl_2__ − δH_αS+10 mM MgCl_2__)^2^ + (0.15*(δN_(ADP or ATP)+10 mM MgCl_2__ − δN_αS+10 mM MgCl_2__)^2^)))^1/2^.^42^ (i) αS ΔCCS induced by 10 mM ATP-Mg *vs.* ATP, ADP-Mg *vs.* ADP and AMP-Mg *vs.* AMP. ΔCCS are plotted *versus* αS residue number and calculated for panels (c), (d), (g) and (i) relative to αS. (j) Cos *θ* profile from CHESPA analysis of 10 mM ATP-Mg binding to αS relative to αS and αS bound to 10 mM ATP, plotted *versus* αS residue number and with a 0.001 ppm cut-off. *θ* angle is between the perturbation vector from αS and 10 mM ATP to αS and 10 mM ATP-Mg and the reference vector of αS to αS and 10 mM ATP. αS spectra in the absence or presence of 10 mM ATP are shown for reference. αS regional charges are shown above plots (c), (d) and (g)–(j), with dark grey boxes representing the imperfect “KTKEGV” repeats.^17^

Our data show that formation of the ATP-Mg complex attenuates but does not eliminate the significant chemical shifts induced by ATP or Mg^2+^ at the N- and C-termini of αS, respectively (Fig. 4a–c). This observation is consistent with the notion that the charge neutralization of both ATP and Mg^2+^ causes ATP-Mg to bind αS less strongly. As such, the increasing formation of ATP-Mg accounts for the concentration-dependent decreases in the C-terminal Mg-induced αS chemical shifts as well as the absence of increasing N-terminal ATP-induced αS residue shifts as more ATP is added to Mg-bound αS (Fig. 4d–f). The sequestration by ATP of Mg^2+^ ions away from αS is further confirmed by CHESPA (ESI Fig. S3†), a type of NMR chemical shift projection analysis summarized in detail by Narayanan *et al.*^33^ Conversely, as increasing concentrations of Mg^2+^ are added to ATP-bound αS, the N-terminal ATP-induced αS chemical shifts exhibit concentration-dependent decreases that are not accompanied by increased C-terminal Mg-induced αS residue shifts, indicating that the de-tuning effect of ATP on Mg^2+^ binding to αS is reciprocal (Fig. 4g). The dynamic interplay between ATP, Mg^2+^, ATP-Mg and αS suggests that ATP could serve as a Mg^2+^ “sink,” buffering its effects on αS.

We next explored Mg^2+^ sequestration by ATP's triphosphate moiety, and its consequences on the amount of free Mg^2+^ available to bind αS, by comparing the effects of ADP *versus* ATP on Mg-bound αS (Fig. 4h).^28^ When considered relative to the Mg-induced αS residue shifts, the significant C-terminal shifts induced by ATP and ADP support our hypothesis that the nucleotides are able to sequester Mg^2+^ away from αS in a phosphate-dependent manner (Fig. 4h). Fig. 4h also shows that the phosphate negative charges are the major driving force of the ATP–αS interaction, since despite the reduced de-tuning effect of ADP on the αS-Mg^2+^ interaction, the reduced number of phosphate groups still hinders ADP's relative association with the αS N-terminus, as evidenced by the absence of increased N-terminal shifts. Hence, our emerging model of an electrostatic, phosphate-driven and Mg^2+^-modulated effect of ATP on αS somewhat differs from previous hypotheses that Mg^2+^ bridges ATP primarily with the αS C-terminus (Fig. 1d).^12^ However, it is possible that the Mg-sequestration and Mg-bridging models are not mutually exclusive and represent two viable mechanisms of ATP–αS interactions, one of which may prevail under diverse experimental conditions.

Considering the pathologically-relevant effect of free Mg^2+^ on αS aggregation, we tested whether an equimolar ATP-Mg “buffer” leaves residual free Mg^2+^ available to interact with αS. We measured the αS chemical shifts in solutions of AMP-Mg, ADP-Mg and ATP-Mg, relative to those induced by AMP, ADP or ATP alone and saw a clear pattern of increasing, Mg-induced C-terminal αS chemical shifts, suggesting that the decreasing numbers of phosphate groups in ATP, ADP and AMP, respectively, leave consecutively more Mg^2+^ available to bind αS (Fig. 4i). Nevertheless, the pattern of C-terminal αS shifts induced by ATP-Mg *versus* ATP indicate that ATP is unable to chelate 100% of equimolar Mg^2+^ away from αS, suggesting that some free ligands in a biologically-relevant ATP-Mg “buffer” can bind αS (Fig. 4i).^28^ This is consistent with our CHESPA results, which show that ATP-Mg accentuates the effect of residual Na^+^ cations on the αS C-terminus by shifting it farther from the αS alone state relative to the ATP-bound state (Fig. 4j).^33^ Meanwhile, Mg^2+^ complexation with ATP shifts the αS N-terminus back toward the unbound state, as evidenced by the predominantly negative cos *θ* CHESPA values, which are consistent with our hypothesis of ATP-Mg sequestering ATP from αS (Fig. 4j).^33^ Given the lack of major ATP- or Mg-induced NAC-region αS chemical shifts (Fig. 3c; 4c and d), we hypothesize that the negative cos *θ* NAC-region CHESPA values report on a perturbation of long-range contacts between αS residues, which have been shown to shield the NAC region.^20,21,33^ We therefore tested this hypothesis using intramolecular Paramagnetic Relaxation Enhancement (PRE) NMR experiments, which report on long-range contacts between the N- and C-termini of αS monomers.^20^

### ATP disrupts long-range electrostatic contacts in αS monomers

**Fig. 5:**
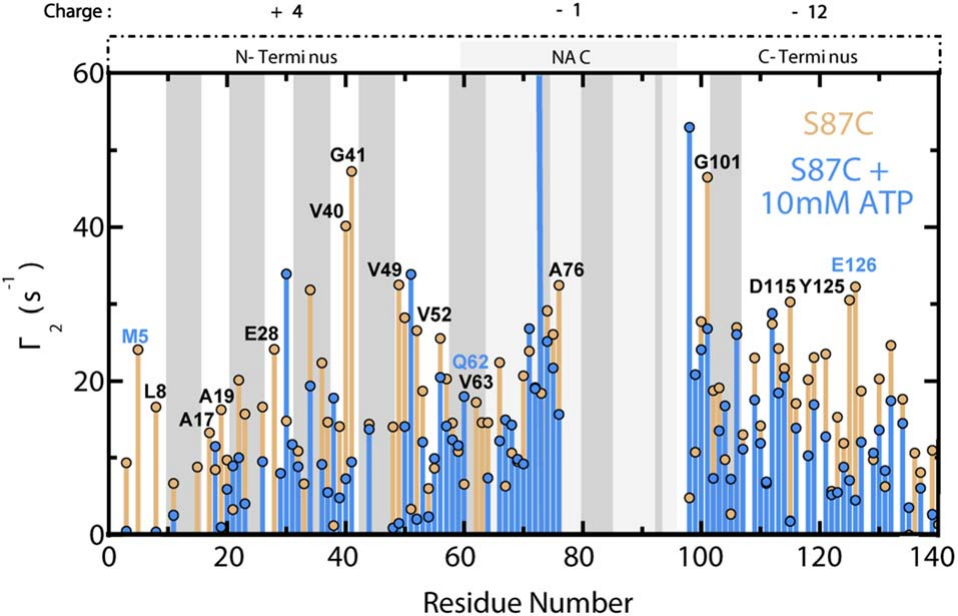
ATP disrupts long-range N- to C-terminal interactions in αS monomers. Residue-specific *Γ*_2_ values for spin-labelled, fresh 120 μM S87C^15^N αS +/− 10 mM ATP. *Γ*_2_ differences between the samples without and with ATP greater than region-specific averages plus one standard deviation are labelled. Blue-labelled residues exhibit significantly shifted ppm values by 10 mM ATP. The 10 residues on either side of 87 are not shown, and the αS regional charges are shown. Grey boxes represent “KTKEGV” αS repeats.^17^

To explore the effect of ATP on long-range N- to C-terminal contacts in αS monomers, we measured residue-specific PRE *Γ*_2_ relaxation rates of spin-labelled S87C αS in the absence and presence of 10 mM ATP (Fig. 5).^20,41^ Our *Γ*_2_ data reveal that ATP causes widespread decreases in residue-specific αS *Γ*_2_ values that are particularly significant for the first ∼50 residues as well as residues 110–140, indicating that these monomer regions are comparatively farther from the S87C spin label in the presence of ATP than in its absence (Fig. 5). Based on these results, we hypothesize that the phosphate-driven targeting of ATP to the αS N-terminus disrupts long-range electrostatic contacts between the N- and C-termini of ‘closed’ αS monomers, causing opening (Fig. 1a).^20,21^ These results offer a viable explanation for the ATP-driven, concentration-dependent shortening of the αS lag time in Fig. 2a, and suggest that ATP acts similarly to other charged species which perturb the electrostatic contacts in αS monomers to expose the hydrophobic NAC region and drive increased αS aggregation.^21–24^

### ATP inhibits the N-terminally-driven interaction between αS monomers and fibrils

**Fig. 6:**
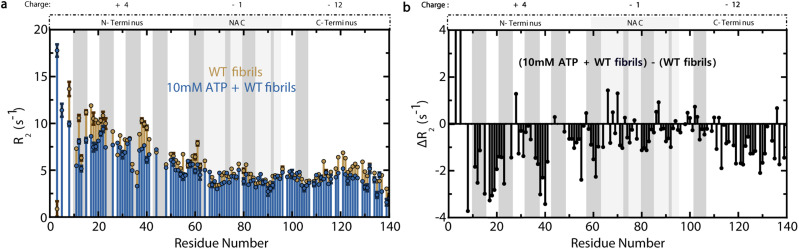
ATP inhibits N-terminally-driven αS monomer–fibril interactions. (a) ^15^N–R_2_ profiles of 250 μM WT αS monomers in the presence of 1.3 mM WT ^14^N fibrils +/− 10 mM ATP. (b) R_2_ differences between the two profiles shown in panel a.

Since secondary nucleation is a major driver of αS aggregation and is electrostatically-driven by the N-terminal residues of αS monomers binding to fibrils, we hypothesized that the inhibition of late-stage αS fibril formation by ATP may involve the suppression of fibril elongation and/or secondary nucleation due to the N-terminal binding of αS monomers by ATP.^9,26,34–37^ To test this hypothesis, we measured residue-specific transverse ^15^N αS R_2_ amide relaxation rates in the presence of late-stage WT αS amyloid fibrils in both the absence and presence of ATP (Fig. 6a). We observed dramatic reductions in N-terminal αS R_2_ rates upon addition of ATP to preformed fibrils (Fig. 6). In fact, the general effect of ATP in the presence of fibrils is a widespread decrease in R_2_ rates that, while most significant in the N-terminus, extends throughout almost the entire αS sequence (Fig. 6b). By contrast, 10 mM ATP does not induce dramatic changes in R_2_ rates of WT αS monomers in the absence of fibrils (ESI Fig. S4†). These experiments were modelled after those of Kumari *et al.*, who showed that the primary interaction site between αS monomers and fibrils is the monomeric N-terminus, which displays concentration-dependent increases in R_2_ relaxation rates with increasing amounts of fibrils.^9^ A significant enhancement of amide relaxation rates is expected if an isotopically-labelled αS monomer residue interacts with unlabelled fibrils.^9^ Therefore, the fact that ATP abolishes the fibril-dependent, N-terminal R_2_ increases of αS monomers suggests that ATP inhibits interactions between αS monomers and fibrils, particularly at the monomer N-terminus.

Since the αS monomer–fibril interactions are predominantly electrostatic, it is likely that the muted interactions in the presence of ATP are due to ATP's N-terminal binding and positive-charge neutralization of αS monomers.^9^ Overall, the inhibition of monomer–fibril interactions and the likely consequent suppression of templated fibril elongation and secondary nucleation provide a viable explanation for the ATP-mediated suppression of αS cross β-sheet fibrils at plateau (Fig. 2b).^9,26^ As secondary nucleation is the dominant mechanism of both αS oligomer and fibril formation under physiologically-relevant quiescent conditions, it is likely that ATP's inhibition of the αS monomer–fibril contacts vital for secondary nucleation may play a role in PD-relevant αS aggregation *in vivo*.^35^

### The PD-related mutations E46K and A53T alter the effect of ATP on αS

**Fig. 7:**
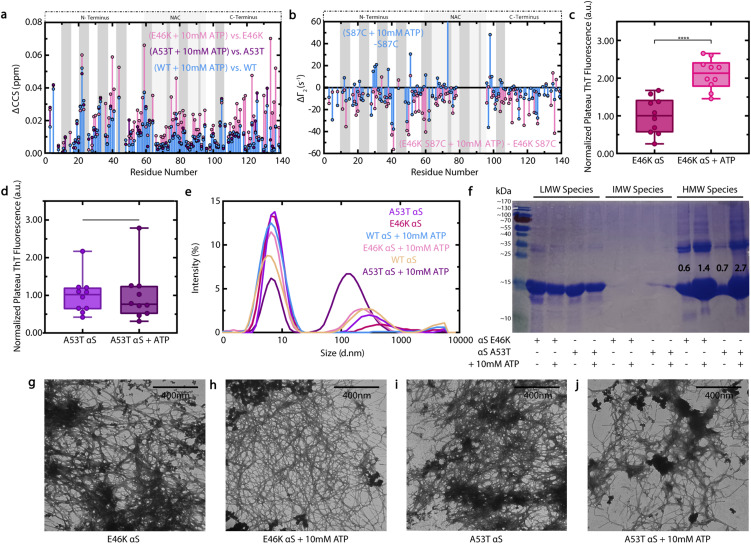
Effect of ATP on E46K and A53T αS. (a) 10 mM ATP-induced ΔCCS of WT, E46K and A53T αS monomers, calculated relative to the corresponding variant alone. (b) PRE-derived *Γ*_2_ value differences induced by 10 mM ATP on spin-labelled, fresh 120 μM ^15^N S87C or S87C E46K αS, calculated relative to the corresponding S87C variant alone. The ten residues on either side of 87 are not shown. (c and d) Well-specific plateau ThT fluorescence measurements of fresh 300 μM E46K (c) or A53T (d) αS in ThT buffer +/− 10 mM ATP, normalized to the average measurement of the corresponding variant protein alone and incubated as for Fig. 2b. (e) DLS measurements of soluble αS from panels (c) and (d) plateau samples, following centrifugation to pellet large aggregates. (f) SDS-PAGE of soluble αS from panels (c) and (d) samples following centrifugation that were subsequently either retained by (IMW) or passed through (LMW) a Pall Nanostep 100 kDa centrifugal filter. Resuspended centrifugation pellets constitute HMW samples. Bands are in reference to PageRuler Prestained Protein Ladder (26616, Thermo Scientific), shown in the left-most lane. Numbers indicate a quantification of the ∼15 kDa monomer band intensities by ImageJ, expressed as the ratio of the HMW/LMW lane monomer bands for each sample. (g–j) Negative stain TEM images of pelleted large aggregates from panels (c) and (d) plateau samples: E46K (g) or A53T (i) αS alone or in the presence of 10 mM ATP (h and j). All scale bars represent lengths of 400 nm.

Finally, we characterized how the effect of ATP on αS is influenced by the presence of PD-related point mutations E46K and A53T, which modify residues targeted by ATP, as shown by chemical shift mapping of WT αS (Fig. 3a–c).^19^Fig. 7a shows that the overall patterns of ATP-induced residue shifts are similar for WT and A53T αS. By contrast, the residue-specific effect of ATP on E46K αS is extremely pronounced relative to WT, with significant chemical shift changes occurring throughout the αS sequence (Fig. 7a). Since E46K αS monomers exhibit increased N- to C-terminal contacts relative to WT, we hypothesized that ATP binding could disrupt these electrostatic contacts and result in widespread changes in overall monomer conformation, leading to the significant chemical shifts shown in Fig. 7a.^24^ Indeed, intramolecular PRE experiments involving S87C E46K αS reveal larger differences in residue-specific *Γ*_2_ values caused by ATP at both the N- and C-termini of E46K αS monomers relative to WT (Fig. 7b). These data suggest that ATP causes a more significant disruption of long-range electrostatic contacts in αS monomers in the presence of the PD-related E46K mutation.^24^ We hypothesize that the presence of an additional positive charge in the E46K αS N-terminus leads to an enhanced electrostatic interaction with ATP, which then causes more significant N-terminal charge neutralization and overall monomer conformational changes toward open states.

To assess whether the residue-specific effects of ATP on E46K and A53T αS monomers lead to altered, pathologically-relevant aggregation, we next measured how ATP influences the ThT fluorescence of E46K and A53T αS at plateau and analyzed the aggregated species by DLS, sodium dodecyl-sulfate polyacrylamide gel electrophoresis (SDS-PAGE) and TEM (Fig. 7c–j, ESI S5†).^9^ Contrary to its inhibitory effect on WT αS at plateau, ATP causes a significant enhancement of E46K αS plateau ThT fluorescence, suggesting that the E46K mutation eliminates the inhibitory effect of ATP on late-stage αS β-sheet fibrillization (Fig. 7c). By contrast, the effect of ATP on A53T αS plateau ThT fluorescence is not significant (Fig. 7d), suggesting nonetheless that the A53T mutation attenuates the inhibitory effect of ATP on αS fibrillization (Fig. 2b). Consistent with the ThT data, our TEM images (Fig. 7g–j) show that E46K and A53T αS fibrils form in both the absence and presence of ATP.

In addition, our DLS data (Fig. 7e) shows that ATP shifts the population of soluble A53T αS away from small, low-molecular-weight species towards intermediately-sized (∼100 nm) aggregates in a manner distinct from its effect on WT. This effect is moreover independently confirmed by SDS-PAGE (Fig. 7f), which shows that the A53T mutation increases the population of High-Molecular-Weight (HMW) assemblies relative to Low-Molecular-Weight (LMW) species. Interestingly, similar mutation-induced DLS and SDS-PAGE changes are observed for E46K αS, albeit less extreme (Fig. 7e and f). Nonetheless, these changes are likely pathologically-significant, as Emin *et al.* recently showed that soluble, small – but not monomeric – αS oligomers induce the greatest release of tumour necrosis factor α, a pro-inflammatory cytokine linked to PD progression, upon addition to mouse microglia.^31^ As the size of αS aggregates correlates inversely with their cytotoxicity, the fact that ATP affects the size distribution of soluble αS aggregates is likely particularly pathologically-relevant.^31^ Overall, our ThT, TEM, DLS and SDS-PAGE data consistently indicate that the A53T and E46K αS mutations markedly inhibit the ATP-induced suppression of intermediate-size and fibrillar assemblies observed for WT αS, consistent with their pronounced early-onset PD phenotype.^19^

## Experimental

Detailed experimental procedures and ESI data are described in the ESI.†

## Conclusions

**Fig. 8:**
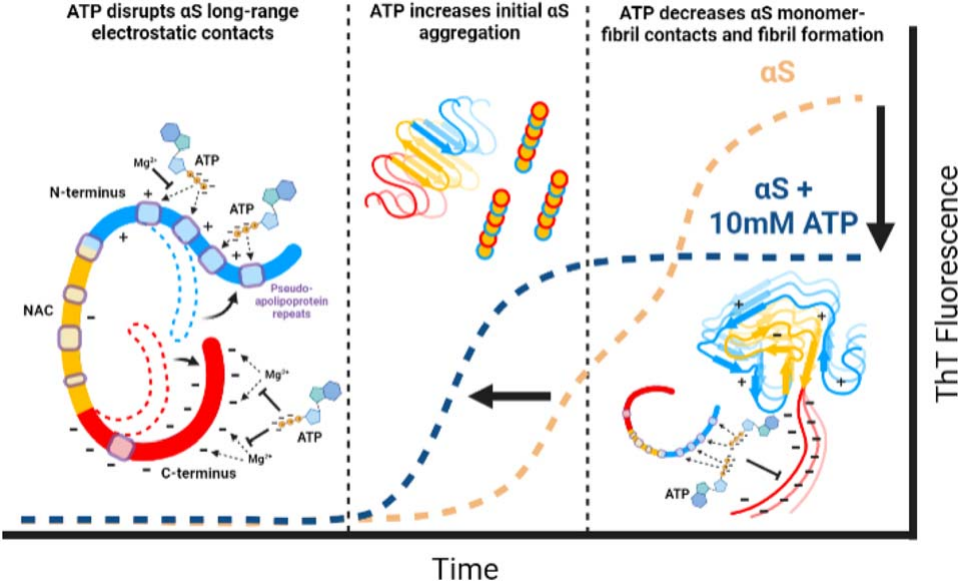
Proposed summary model of αS/ATP interactions and their effects.

Here we provide a residue-resolution picture of the αS–ATP interactions and show how ATP influences the aggregation of WT αS as well as its PD-related variants E46K and A53T.^19^ Our results reveal that ATP directly binds αS *via* its triphosphate moiety and elicits a direct and bimodal effect on WT αS aggregation by disrupting long-range electrostatic contacts in monomers to shorten the aggregation lag time (Fig. 8). ATP also inhibits late-stage cross β-sheet αS fibril formation as well as the αS monomer–fibril interactions needed for secondary nucleation (Fig. 8).^9^ We also show that Mg^2+^ inhibits the ATP–αS interactions and *vice versa*, suggesting that ATP-Mg serves as a “sink” to buffer the amount of free ATP and Mg^2+^ available to interact with αS (Fig. 8). This study provides a viable molecular mechanism to explain the hydrotrope-like function of ATP as it relates to a prototypical amyloid system.^43–49^ As well, our results reveal that the loss of this novel function of ATP caused by known αS pathogenic mutations offers a new perspective on early-onset PD progression.^19^

## Author contributions

EKH prepared samples, designed, executed, and analysed all NMR/ThT/DLS/TEM experiments and wrote the manuscript. JH and RA contributed to the acquisition and analysis of select NMR, ThT and TEM data. GM contributed to the experimental design, the analysis of the data, and the writing of the manuscript.

## Conflicts of interest

There are no conflicts to declare.

## Supplementary Material

SC-014-D3SC03612J-s001
