# Supplementary material for: Toward a molecular mechanism for the interaction of ATP with alpha-synuclein
Source: Chem Sci. 2023 Aug 26;14(36):9933–42. doi: 10.1039/d3sc03612j (PMC10510630; doi:10.1039/d3sc03612j)

Electronic Supplementary Material (ESI) for Chemical Science.
This journal is © The Royal Society of Chemistry 2023

Supplementary Information for

Toward a Molecular Mechanism for the Interaction of ATP with Alpha-Synuclein

Evelyn Rose Kamski-Hennekam,[b] Jinfeng Huang,[b] Rashik Ahmed,[b] and Giuseppe Melacini*[a,b]

[a] Dr. G. Melacini
Department of Biochemistry and Biomedical Sciences
McMaster University
Hamilton, ON, L8S 4M1
E-mail: melacin@mcmaster.ca

[b] E.R. Kamski-Hennekam, J. Huang, Dr. R. Ahmed, Dr. G. Melacini
Department of Chemistry and Chemical Biology
McMaster University
Hamilton, ON, L8S 4M1

Table of Contents

p. 1-3 Experimental Procedures
p. 4-5 Supplementary Figures

Experimental Procedures

Recombinant Alpha-Synuclein Expression and Purification

Expression and purification of recombinant WT, S87C, A53T, E46K and E46K S87C αS protein was accomplished by following a published protocol with slight modifications.[34] Briefly, *E. coli* BL21(DE3) cells transformed with a pT7-7 plasmid containing the αS sequence were grown at 37 ºC with shaking either in Luria–Bertani medium (for preparation of unlabeled protein for ThT and fibril experiments) or isotope-enriched M9 minimal media containing 1 g/L of ^{15}N-ammonium chloride (for preparation of ^{15}N-labeled protein) and, in both cases, 100 µg/mL of ampicillin. After reaching an OD600 of ~0.6-0.8, αS expression was induced with 100 µM isopropyl β-D-1-thiogalactopyranoside (IPTG) at 37 °C with shaking for 4-5 hrs and cells were pelleted by centrifugation at 3836 X g. Cell pellets were stored at -80 ºC until use. After resuspending cell pellets in lysis buffer (10 mM Tris-HCl, 1 mM EDTA, 1 mM AEBSF protease inhibitor 101500 from EMD Millipore, pH 8), cells were lysed via three freeze-thaw cycles followed by sonication and heating to ~100 °C for 20 min. Cell lysates were centrifuged at 12326 X g for 1 hr to pellet cell debris, at which point 10 mg/mL streptomycin sulfate was slowly added to the supernatant, which was then stirred for 20 min at 4 °C followed by further centrifugation at 20376 X g for 20 min to pellet nucleic acids. Alpha-synuclein protein was precipitated from the supernatant by adding, extremely slowly, 360 mg/mL ammonium sulfate and stirring for 20 min at 4 °C. After centrifuging the mixture at 20376 X g for 20 min, the resulting pellet was resuspended in 25 mM Tris-HCl, pH 7.7, placed in 3.5 kDa-cutoff dialysis tubing and dialyzed against 5 L of the same buffer for 2 hrs to remove any remaining ammonium sulfate. The dialyzed protein was then filtered with a 0.2 µM filter and loaded onto an anion exchange column (HiTrap Q Sepharose high performance, GE healthcare) and eluted with a 0−600 mM NaCl step gradient. Purified αS eluted at ~300 mM NaCl and was concentrated using a 10 kDa Amicon centrifugal filter unit, filtered as before and then loaded onto a Hiload 16/600 Superdex 200 column pre-equilibrated with either milliQ H_2O, NMR buffer (50 mM Hepes, 5% D_2O, pH 7.4), ThT buffer (20 mM K_2HPO_4, 5 mM KH_2PO_4, 100 mM KCl, 200 µM EDTA, 0.05% NaN_3, pH 7.4) or fibril buffer (1X PBS, 0.05% NaN_3, pH 7.4).[36,37] The eluted protein was then concentrated using another 10 kDa Amicon centrifugal filter unit. The final protein concentration was confirmed by A280 measurements using an extinction coefficient of 5600 M^{-1} cm^{-1}.[36]

Preparation of WT αS fibrils

Formation of late-stage αS fibrils was accomplished by incubating 1.5 mL Eppendorf tubes containing 500 µL aliquots of 500 µM monomeric αS protein prepared in fibril buffer at 37 ºC in a rotating incubator at approximately 200 rpm for 2-3 weeks, following the general protocol of Kumari *et al.*[9] Fibrils were then pelleted by ultracentrifugation at 121968 X g for 1 hr at 4 ºC; the supernatant was removed, and the ultracentrifugation was repeated after resuspending the fibrils in 1.2 mL of NMR buffer. Pellets were stored at 4 ºC for not more than two weeks until use, whereupon the fibril pellet was washed two times with NMR buffer to remove salt and resuspended until homogenous in NMR buffer. The fibril concentration was approximated using the equivalent monomer concentration.

Solution NMR Spectroscopy

All solution NMR spectra were recorded using a Bruker AV 700 spectrometer equipped with a TCI cryo-probe at approximately 10 ºC and were analyzed with TopSpin 4.0.9., and Sparky using Gaussian line-fitting. Additional details are discussed below.

^{1}H-^{15}N Heteronuclear Single Quantum Coherence (HSQCs)

The residue-specific effects of ATP and ATP analogs on αS monomers were monitored by two-dimensional ^{1}H-^{15}N HSQC NMR experiments with water suppression using a 3-9-19 pulse sequence with gradients, recorded with a recycling delay d_1 of 1s, 8 scans, and 4K (t_2) and 300 (t_1) complex points for spectral widths of 16.2 ppm (^{1}H) and 35.0 ppm (^{15}N), respectively. Chemical shift changes were measured through Gaussian fitting of the peaks in Sparky. Stock solutions of AMP, ADP, ATP, Sodium Triphosphate (purchased from Sigma-Aldrich) and salts $MgCl_2$ and NaCl (purchased from Alfa Aesar and Fisher Scientific) were prepared on ice in NMR buffer with pH adjusted to 7.4 using NaOH and HCl. ATP-Mg was prepared on ice by dissolving equimolar amounts of ATP and $MgCl_2$ into NMR buffer and incubating overnight on a rocker at 4 ºC before adjusting the pH to 7.4 as above. Samples of fresh, 120 μM ^{15}N WT αS monomers in NMR buffer were prepared in 5 mm NMR tubes in the presence or absence of increasing concentrations of ATP, ATP-Mg, $MgCl_2$ or ATP analogs. For experiments with Mg- or ATP-bound αS, samples of αS in NMR buffer with either 10 mM $MgCl_2$ or ATP were incubated overnight on a rocker at 4 ºC before the addition of the other additive. Compounded chemical shifts were calculated using the formula ΔCCS = $(0.5*((\delta H_{Additive} - \delta H_{No\ Additive})^2 + (0.15*(\delta N_{Additive} - \delta N_{No\ Additive})^2)))^{1/2}$. Deviations from this formula are outlined in specific figure captions. In specific cases, the chemical shift data were fitted to the one-site specific binding model in GraphPad Prism 8.4.2., with the model-calculated approximate K_ds shown and errors either estimated as per the similar work of Selvaratnam *et al.* or represented by the standard deviations of all residues in a given sample (for Figure 3f).[38]

CHEmical Shift Projection Analysis (CHESPA)

The CHESPA analysis involved ^{1}H-^{15}N HSQCs acquired as described above for four samples: fresh 120 μM ^{15}N WT αS in NMR buffer in the absence or presence of 10 mM ATP, ATP-Mg or $MgCl_2$. The reference vector was defined as that from the αS sample to the sample of αS with 10 mM ATP, and the perturbation vector was defined as that from the sample of αS with 10 mM ATP to the sample of αS and 10 mM ATP-Mg. The angle between the perturbation and the reference vector is denoted as θ, and is defined as per equation 6 in Boulton *et al.*[39] The CHESPA analysis was performed in Sparky using a scaling factor of 0.15 for the ^{15}N dimension and a cut-off of 0.001 ppm. A detailed description of the CHESPA analysis is reported by Shao *et al.*[40]

Pairwise Chemical Shift Projection Analysis

To assess whether the shifts of WT αS ^{1}H-^{15}N HSQC cross-peaks induced by 10 mM ATP are consistently in one relative direction, residue-specific displacement vectors similar to equation 4 from Ahmed *et al.* were defined, with "holo" and "apo" representing samples in the presence and absence of ATP, respectively.[32] Cos theta angles between displacement vectors of generic residues *i* and *j* were computed through the normalized dot product as in equation 5 from Ahmed *et al.*[32] Residue pairs with $\cos\theta_{ij}$ values greater than 0.97 were then visualized in a 140 X 140 matrix.

Intramolecular Paramagnetic Relaxation Enhancement (PRE) to Probe αS Monomer Unfolding in the Presence of ATP

The effect of ATP on long-range electrostatic contacts between the N- and C-termini of αS monomers was probed using 120 μM samples of monomeric ^{15}N WT S87C or E46K S87C αS spin-labeled with non-acetylated MTSL (S-(1-oxyl-2,2,5,5-tetramethyl-2,5-dihydro-1H-pyrrol-3-yl)methyl methanesulfonothioate) in the absence or presence of 2 mM ascorbic acid. Briefly, samples were prepared by dissolving lyophilized αS in NMR buffer to a final concentration of 1.256 mM, upon which a 6 X molar excess of DTT was added and the mixture was incubated on a room-temperature rocker in the dark for approximately 30 min before being loaded onto a 7 kDa MWCO Zeba spin desalting column (Thermo 89891). The concentration of eluted monomeric protein was confirmed as before, MTSL was added at a 10 X molar excess and the protein mixture was incubated on a room-temperature rocker in the dark for four hours before being run through a Hiload 16/600 Superdex 200 column equilibrated with NMR buffer. The eluted protein was concentrated using a 10 kDa Amicon centrifugal filter unit and the final protein concentration was confirmed as before. NMR samples were prepared using freshly concentrated protein in 5 mm NMR tubes and paramagnetic relaxation enhancement rates were measured with d30 delays of (t_a) 4.1 ms and (t_b) 10 ms. The read-out HSQC experiments were recorded with 300 (t_1) and 2048 (t_2) complex points and spectral widths of 35.00 and 16.23 ppm for the ^{15}N and ^{1}H dimensions, respectively. Thirty-two scans were recorded with a recycle delay of 1 s. Ascorbic acid was added to each sample to reduce the MTSL spin label and the protein was incubated for 1 hr at 283 K before the above NMR experiments were repeated. Γ_2 relaxation was calculated based on the following equation, using peak intensities from the four HSQC spectra acquired for each sample: $\Gamma_2 = (1/(t_b - t_a))*(\ln[\{I_{reduced,tb}*I_{oxidized,ta}\}/\{I_{reduced,ta}*I_{oxidized,tb}\}])$, based on equation 5 from Iwahara *et al.*.[41]

Transverse ^{15}N Amide Relaxation R_2 Experiments to Characterize WT αS Monomer-Fibril Interactions in the Presence of ATP

Changes in αS residue relaxation rates upon binding to mature, late-stage fibrils were monitored via transverse ^{15}N amide relaxation R_2 experiments involving fresh 250 μM αS monomers prepared in NMR buffer in the presence or absence of both 1.325 mM (5.4-fold molar excess) unlabeled WT α-Syn fibrils and/or 10 mM ATP. ^{15}N transverse relaxation rates (^{15}N-R_2) were measured using a pseudo 3D pulse sequence with water flip-back, heat compensation and sensitivity enhancement. The total CPMG lengths were 31.36, 62.72, 94.08, 125.44, 156.88 and 188.18 ms using a τ delay (half the time between 180º pulses) of 900 μs.[42] Spectra were acquired with 2048 (t_2) and 300 (t_1) points, 32 scans and recycle delays of 1 s.

ThT Fluorescence

The effects of ATP and ATP analogs on both initial and late-stage αS aggregation were monitored by ThT fluorescence measurements of fresh, 300 µM monomeric αS protein samples prepared in ThT buffer in the absence or presence of additives. Stock samples were prepared in 1.5 mL Eppendorf tubes on ice before being aliquoted into each of four wells of black, Greiner half-volume, glass-bottomed 96-well plates containing one 3 mm glass bead per well. Sample wells contained 110 µL solution, while blank wells and buffer dams contained 110 µL and 300 µL buffer, respectively. Plates were sealed with a plastic sheet as well as a lid and ThT fluorescence measurements were recorded from the plate bottom on a BioTek Instruments Cytation 5 Cell Imaging Multi-Mode plate reader set with extended gain and excitation and emission at 448 and 482 nm. For experiments monitoring the entire ThT time profile, plates were incubated in the reader for either 85 or 47 hours at 37 ºC with 30 s orbital shaking prior to each read, spaced six minutes apart. Data are reported as the average fluorescence of the four wells for each condition at each timepoint. For plateau experiments, after taking baseline initial readings every 3-5 min for 30 min with 30 s orbital shaking prior to each read, plates were placed in a 37 ºC shaker at 150 rpm for 72 hrs before being incubated in the 37 ºC reader for 20 additional hours at plateau, with measurements also taken every 3 min as before. Data are reported as the 20-hour average fluorescence reading for each well of each condition at plateau, with error bars representing the standard deviation of all well averages for each condition, calculated in GraphPad Prism 8.4.2. For the data normalization, the average ThT measurements in Figures 2a and c are normalized to the final measurements of the αS and αS + 10 mM $MgCl_2$ samples, respectively; for Figures 2b and d, the well-specific plateau ThT measurements are normalized to the average measurements of the αS and αS + 10 mM $MgCl_2$ samples, respectively. For the data in Figure 2c, we chose conditions similar to those used by Nishizawa *et al.*; namely we first added 10 mM $MgCl_2$ to WT αS monomers and subsequently added equimolar ATP.[12] To assess the statistical significance of ThT fluorescence differentials, we used the two-tailed, unpaired t-test assuming Gaussian distributions and that both compared populations have the same standard deviation. A difference was deemed significant if it exhibited a p-value of less than 0.05.

Dynamic Light Scattering (DLS)

DLS was used to probe the effect of ATP and ATP analogs on the formation of intermediately-sized, soluble αS aggregates. The above plateau ThT samples were diluted 2:1 with ThT buffer and ultracentrifuged at 121968 X g and 4 ºC for 35 min. The supernatant was used for DLS measurements in 40 µL low-volume plastic cuvettes (ZEN0040) and measured at 10 ºC using 5 runs and 12 measurements per run. DLS was performed using a Zetasizer Nano ZS Instrument (Malvern Instruments, Malvern, UK). Autocorrelation functions were accumulated for 20 min with 20 s per measurement and 120 s of pre-equilibration time, at an angle θ of 173° and using a 4 mW He−Ne laser operating at a wavelength of 633 nm. The particle diameter detection limit was 0.3 nm−10 µm. The viscosity value for water was used in the analysis of all measurements.

Negative Stain Transmission Electron Microscopy (TEM)

To validate the ThT results and confirm the presence of fibrils, the plateau ThT samples were diluted 20 X using milliQH_2O and 3-5 µL drops of the diluted mixtures were spotted on Formvar-coated Cu/Pd grids (Electron Microscopy Sciences, G200-CP and RT 15800) for 5-7 min. After blotting excess sample with filter paper, the grids were stained with equal volumes of 1% uranyl acetate for 1 min. Grids were loaded in a room-temperature holder and introduced into a JEOL 1200-EX electron microscope operated at 80 kV and imaged at 40,000 X direct magnification. All images were acquired with an AMT XR-41 side-mount cooled 4-megapixel format CCD camera.

Sodium Dodecyl-Sulfate Polyacrylamide Gel Electrophoresis (SDS-PAGE)

To analyze the effect of ATP on the relative amounts of low-molecular-weight (LMW), intermediate-molecular-weight (IMW) and high-molecular-weight (HMW) aggregates of E46K and A53T αS, 500 µL aliquots from the aforementioned plateau ThT sample supernatants used for DLS were centrifuged at 14,000 X g for 20 min at 4 ºC using a Pall Nanostep 100 kDa centrifugal filter. The flow-through was designated the LMW sample. The retained material was resuspended in 50 µL ThT buffer as the IMW sample. The DLS ultracentrifuge pellet was resuspended in 100 µL milliQH_20 as the HMW sample. Each LMW and HMW sample was mixed at a 1:1:3 ratio with Laemmli buffer and ThT buffer, while each IMW sample was mixed at a 4:1 ratio with Laemmli buffer for SDS-PAGE. The IMW sample ratio was different from that for LMW and HMW samples to enable visualization of lowly-populated species on the gel. These samples were heated to ~100 ºC for 5 min and separated on an 18% SDS-PAGE gel at 150 V. Gels were stained for 10 min at room temperature following 30 s of heating, then de-stained overnight and imaged. To quantify the relative amounts of HMW versus LMW aggregates for each condition, the intensities of each monomer band at approximately 15 kDa were estimated in ImageJ and the HMW/LMW intensity ratios were calculated.

Supplementary Figures

Supplementary Figure 1. Negative-stain TEM images of pelleted large aggregates at plateau. Scale bars represent lengths of 400 nm. Other sample details are as in Figure 2b.

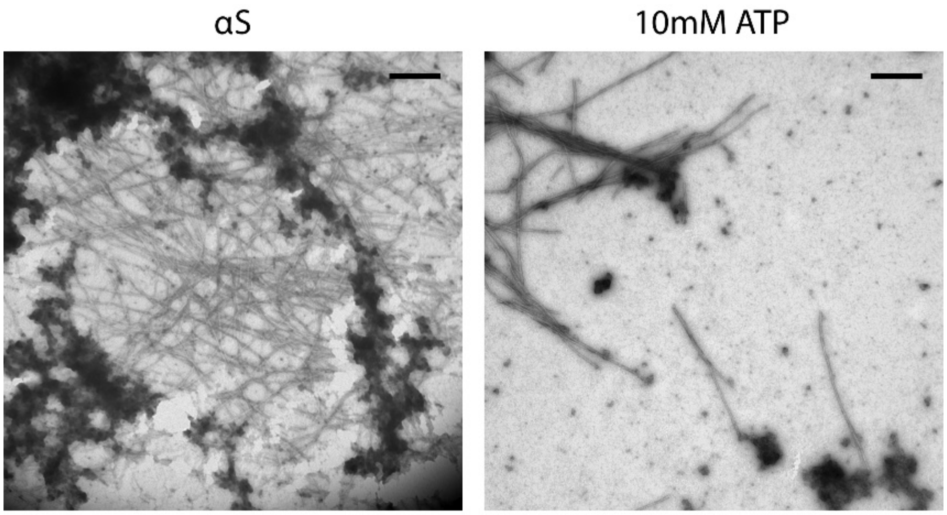

Supplementary Figure 2. ATP suppresses the formation of intermediate-size, soluble αS aggregates. DLS intensity measurements of soluble αS recovered from specific samples from Figure 2b at plateau and following centrifugation to pellet large aggregates.

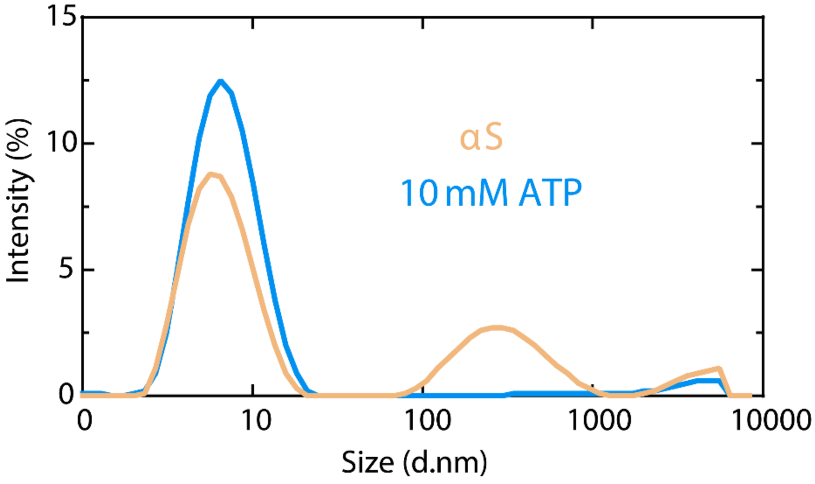

Supplementary Figure 3. CHESPA analysis of the effect of ATP addition to a complex of αS and Mg^{2+}. Residue-specific cosθ profile of CHESPA analysis based on the reference and perturbation vectors shown in the figure inset. The charges of labeled αS regions are shown above plots with dark grey boxes representing the imperfect "KTKEGV" αS repeats.[17]

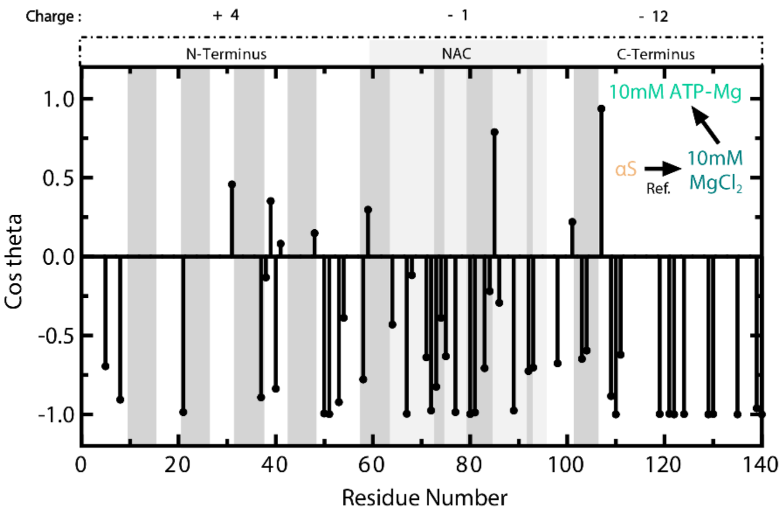

Supplementary Figure 4. ATP does not significantly affect chemical exchange dynamics of αS monomers. ^{15}N-R_2 profiles of 250 μM WT αS monomers +/- 10 mM ATP.

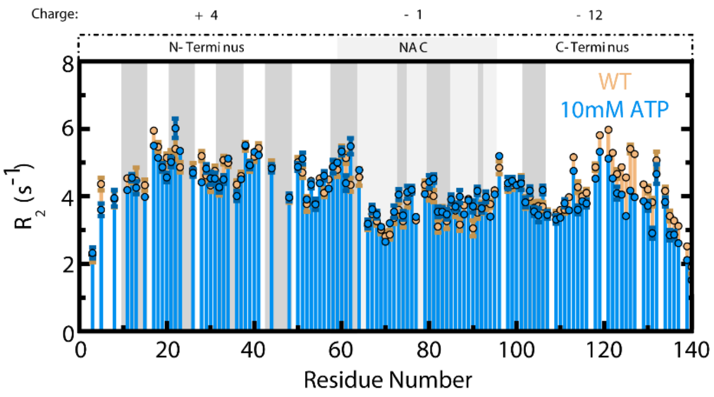

Supplementary Figure 5. Schematic for analysis of aggregated E46K and A53T αS. Plateau ThT samples were diluted 2:1 in ThT buffer before being ultracentrifuged at 121968 x g for 35 min at 4 ºC. The supernatant was used for DLS and the pellet was designated as the HMW sample. Following DLS, the sample was centrifuged in a 100 kDa centrifugal filter at 14,000 X g for 20 min at 4 ºC. The flow-through was designated the LMW sample, while the retained material was designated the IMW sample. Blue spheres represent soluble oligomers, whose actual structure remains elusive.

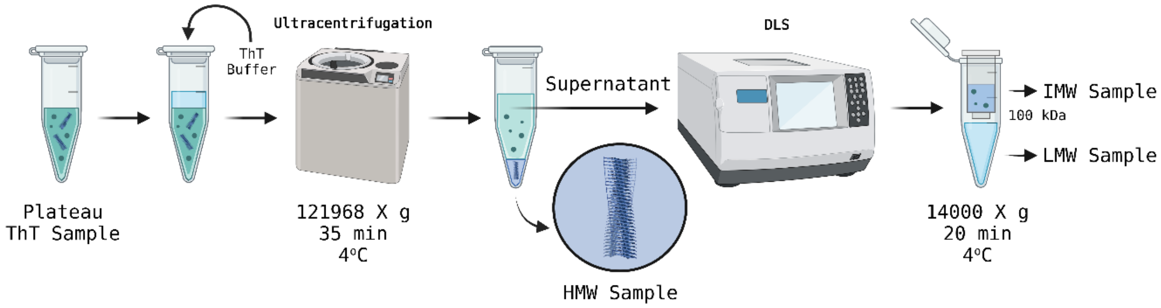

Supplement: SC-014-D3SC03612J-s001 [file SC-014-D3SC03612J-s001.pdf]
